# Dexamethasone Is Not Sufficient to Facilitate Tenogenic Differentiation of Dermal Fibroblasts in a 3D Organoid Model

**DOI:** 10.3390/biomedicines11030772

**Published:** 2023-03-03

**Authors:** Niklas Kroner-Weigl, Jin Chu, Maximilian Rudert, Volker Alt, Chisa Shukunami, Denitsa Docheva

**Affiliations:** 1 Laboratory for Experimental Trauma Surgery, Department of Trauma Surgery, University Hospital Regensburg, 93053 Regensburg, Germany; 2 Department of Orthopedic Surgery, The Second Hospital of Dalian Medical University, Dalian 116023, China; 3 Department of Musculoskeletal Tissue Regeneration, Orthopedic Hospital König-Ludwig-Haus, University of Würzburg, 97074 Wuerzburg, Germany; 4 Department of Molecular Biology and Biochemistry, Biomedical Sciences Major, Graduate School of Biomedical and Health Sciences, Hiroshima University, Hiroshima 734-8553, Japan

**Keywords:** 3D organoids, dermal fibroblasts, dexamethasone, scaffold-free, tenogenic differentiation, tendon tissue engineering

## Abstract

Self-assembling three-dimensional organoids that do not rely on an exogenous scaffold but maintain their native cell-to-cell and cell-to-matrix interactions represent a promising model in the field of tendon tissue engineering. We have identified dermal fibroblasts (DFs) as a potential cell type for generating functional tendon-like tissue. The glucocorticoid dexamethasone (DEX) has been shown to regulate cell proliferation and facilitate differentiation towards other mesenchymal lineages. Therefore, we hypothesized that the administration of DEX could reduce excessive DF proliferation and thus, facilitate the tenogenic differentiation of DFs using a previously established 3D organoid model combined with dose-dependent application of DEX. Interestingly, the results demonstrated that DEX, in all tested concentrations, was not sufficient to notably induce the tenogenic differentiation of human DFs and DEX-treated organoids did not have clear advantages over untreated control organoids. Moreover, high concentrations of DEX exerted a negative impact on the organoid phenotype. Nevertheless, the expression profile of tendon-related genes of untreated and 10 nM DEX-treated DF organoids was largely comparable to organoids formed by tendon-derived cells, which is encouraging for further investigations on utilizing DFs for tendon tissue engineering.

## 1. Introduction

Pathologies of the musculoskeletal system represent a major burden on the global healthcare system and can decrease the quality of life of the affected patients drastically [1,2,3]. It has been estimated that approximately 45% of the 32 million musculoskeletal injuries reported every year in the USA alone involve tendon, ligament, and joint capsular lesions [4,5]. Unfortunately, the natural process of tendon healing, as well as conservative and surgical treatments, frequently fail to produce the desired long-term clinical outcome in tendon injuries [6,7,8,9,10] and, therefore, there is a significant demand for designing and probing novel therapeutic strategies.

Over the last few years, the engineering of functional connective tissues has made considerable progress and has built a strong foundation for developing models with clinical relevance [11]. These efforts mostly rely on the combination of three key components: cells, growth factors, and carriers [12]. However, scaffold-free approaches have also been explored, which hold several advantages over strategies utilizing synthetic or biological carrier materials. Here, naturally occurring cell-to-cell and cell-to-matrix interactions are formed, thus eliminating the need for an exogenous scaffold [13,14]. A popular example of a scaffold-free method is the so-called pellet culture model, which is prominently used for in vitro studies of chondrogenesis [15]. Our study group previously reported a self-assembling three-dimensional (3D) organoid model comprising three consecutive steps (expansion, stimulation, and maturation) that was used in the field of tendon and ligament tissue engineering using various cell types, such as tendon stem/progenitor cells (TSPCs), bone marrow-derived mesenchymal stromal cells (BMSCs), periodontal ligament cells, and dental follicle cells [16,17,18].

Currently, several cell types are reported to undergo tenogenic differentiation, but certain disadvantages remain. For example, BMSCs can induce ectopic bone formation, embryonic stem cells can be tumorigenic and are associated with ethical concerns, and safety and efficacy risks exist for reprogrammed/genetically engineered cells [19]. The use of autologous tendon-derived cells, such as tendon stem/progenitor cells or tenocytes, is burdened by the difficulty to obtain them due to comorbidity (using and thus damaging another tendon serving as a cell source) or compromised quality of the primary cells (using the diseased tendon itself). Dermal fibroblasts (DFs) display a high similarity to tenocytes (TCs) at a transcriptional level and show propensity for tenogenic differentiation when appropriately stimulated [19]. It has been reported that DFs and TCs have comparable cell phenotype and behavior in vitro, including production of extracellular matrix (ECM) proteins, such as collagen I (COL1) and III and proteoglycans, as well as ECM remodeling [20]. In addition, gaining, isolating, and propagating autologous adult human DFs is easier than other cell sources and avoids ethical and safety issues. Hence, DFs represent an attractive cell type for testing tenogenic formation due to their pro-tenogenic potential and the comparable low effort and risks for harvesting them via minimally invasive procedures, such as a skin punch biopsy [21,22].

To date, the tendon field is lacking a widely accepted, standardized differentiation protocol, which is in contrast to bone, cartilage, and fat tissue differentiation. Similar to in vitro chondrogenesis using 3D pellet culture [23], tenogenic differentiation might require more complex stimulation than osteogenic and adipogenic differentiation. Therefore, this study focused on the investigation of dermal fibroblast (DF) differentiation towards tendon lineage in vitro by implementing the above mentioned 3D organoid model. The synthetic glucocorticoid dexamethasone (DEX) has been shown to reduce cell proliferation and influence the ECM production of DFs and tendon cells [24,25,26,27]. Moreover, DEX is an indispensable factor used in osteogenic, chondrogenic, and adipogenic differentiation protocols, suggesting that it might also support the differentiation of cells into tenogenic lineage [28,29,30]. Hence, the main aim of this study was to evaluate the effect of DEX on DF tenogenic differentiation using a three-step protocol for organoid formation and maturation. We hypothesized that an application of DEX onto DFs in the 3D organoid model will facilitate a tendon-like phenotype of the DF organoids. Based on literature sources, three different concentrations of DEX, namely 10, 100, and 1000 nM, which had been reported to influence cell proliferation without exhibiting cytotoxicity, were chosen [24,31,32,33]. Here, firstly DFs were exposed to the above DEX doses during the second two-dimensional (2D) stimulation step of the organoid protocol, with the goal of preventing excessive cell proliferation whilst also inducing tenogenic differentiation of the cells. Thereafter, the obtained organoids were investigated at the beginning and end of the 3D maturation step in terms of size, tissue morphology, cell cytoskeletal organization, DNA content, cell proliferation and apoptosis, and multiple gene expression at the mRNA level.

## 2. Materials and Methods

### 2.1. Cell Culture

Normal human dermal fibroblasts (NHDFs) (three different donors, n = 3) were commercially purchased (PromoCell, Heidelberg, Germany) and cultivated in low-glucose DMEM (Thermo Fischer Scientific, Waltham, MA, USA), supplemented with 10% FBS and 5 µg/mL human insulin (both, PAN Biotech, Aidenbach, Germany), 1 ng/mL FGF (Pepro Tech, Hamburg, Germany) and 1% penicillin/streptomycin (Gibco, Karlsruhe, Germany). As the control, human biceps tendon cells (HBTCs) were isolated according to Kohler et al. [34] from one donor (n = 1) (under Ethical Grant No. 18-985-101 of the Ethics Committee of the University of Regensburg). HBTCs culture medium was DMEM/F-12 (Gibco), 10% FBS (PAN Biotech), 1 × MEM Amino Acids Solution (Thermo Fischer Scientific), 1% L-ascorbic acid 2-phosphate (stock 0.05M) (Sigma-Aldrich, St. Louis, MO, USA), and 1% penicillin/streptomycin (Gibco). Both cell types were kept in a humidified incubator at 37 °C and 5% CO_2_. Medium was changed three times per week, and cells were used for experiments in passages 2–5.

### 2.2. Flow Cytometry Analysis (FACS)

One representative donor of NHDFs (*n* = 1) underwent FACS analysis of classical MSC versus hematopoietic cell markers. Cells were washed in PBS, trypsinized, centrifuged, and resuspended in PBS. Then, 3 × 10^5^ cells were incubated with a positive cocktail (consisting of mouse anti-human CD73 antibody conjugated to allophycocyanin (APC), mouse anti-human CD90 antibody conjugated to fluorescein isothiocyanate (FITC) and mouse anti-human CD105 antibody conjugated to PerCP-Cy5.5-conjugated) and a negative cocktail (consisting of mouse anti-human CD34, CD11b, CD19, CD45 and HLA-DR antibodies conjugated to phycoerythrin (PE)). Positive isotype control cocktail (consisting of mouse APC-conjugated, FITC-conjugated and PerCP-C 5.5-conjugated IgG1 antibodies) and PE-conjugated negative isotype control cocktail were also used. All of the above antibodies were purchased from Becton–Dickinson, San Jose, CA, USA. After 45 min antibody incubation on ice, cells were washed, centrifuged and resuspended again in 0.5 mL 1% FCS in PBS. Then, 2 × 10^4^ events per sample were analyzed on a BD FACSCanto II Flow Cytometry System (Becton–Dickinson), measuring forward scatter (FSC) light, side scatter (SSC) light, APC, FITC, PerCP-Cy5.5, and PE emitted fluorescence. Data were analyzed with Flowing software 2.5.1 (http://flowingsoftware.btk.fi, URL (accessed on 21 August 2019), Turku Bioscience, Turku, Finland).

### 2.3. Three-Dimensional Organoid Model

A previously established three-step protocol (2D expansion, 2D stimulation and 3D maturation) for organoid formation and maturation was implemented [17,18]. NHDFs and HBTCs were plated in cell culture petri dishes (10 cm diameter; Falcon, Corning, NY, USA) at a density of 8 × 10^4^ cells/cm^2^, receiving low-glucose DMEM, 10% FBS, 1 × MEM amino acids and 1% penicillin/streptomycin. After reaching full confluency within seven days, the medium for the 2D stimulation stage was changed to high-glucose DMEM (Gibco) supplemented with 10% FBS, 1 × MEM amino acids, 50 µg/mL ascorbic acid and 1% penicillin/streptomycin, which was administered for 14 days. In addition, different concentrations of DEX (10, 100 and 1000 nM; Sigma-Aldrich) were added to the media of the NHDF experimental groups. The NHDF and HBTC control groups did not receive DEX. On the first day of the 3D maturation stage, cell monolayers were gently detached from the bottom of the dish by cell scraper and rolled into 3D rod-like organoids. These were subsequently transferred into a non-adhesive culture dish (10 cm diameter; Sarstedt, Nümbrecht, Germany) and fixed by pins (EntoSphinx, Pardubice, Czech Republic) with manual stretching of approx. 10% axial elongation. The formed organoids were cultured for 14 days in high-glucose DMEM, containing the same ingredients as before with the addition of 10 ng/mL TGF-β3 (Anprotec, Bruckberg, Germany). DEX was not added during the 3D maturation stage. The wet weight of each organoid was measured right after organoid formation (d0), as well as after 14 days (d14) during organoid harvest. In total, 60 NHDF organoids (n = 3 donors, with 5 different organoids/donor, resulting 15 organoids/group) and 5 HBTC organoids (*n* = 1, 5 organoids) were generated. Per NHDF donor, the 5 organoids were used as follows: 1 organoid for DNA quantification right after organoid formation (d0), 2 organoids (d1 and d14) for histological analyses (e.g., H&E, F-actin, Ki-67 and TUNEL staining), and 2 organoids for RNA isolation (d1 and d14). For the HBTC donor, 3 organoids were used for RNA and 2 were stored.

### 2.4. DNA Quantification Assay

To compare cell abundance in the organoids at the end of DEX-treatment, the Quant iT PicoGreen dsDNA Assay Kit (Thermo Fischer Scientific) was used according to the manufacturer’s instructions. NHDF organoids from each experimental group (0, 10, 100, 1000 nM DEX) were collected at d1 (n = 3 donors) of the 3D maturation step, snap-frozen and stored at −80 °C. In preparation for the measurement, the organoid samples were first digested overnight at 60 °C using 150 µg/mL papain (Sigma-Aldrich), 6 mM L-cysteine and 6 mM Na_2_EDTA in PBS with adjusted pH 6. The resulting solution was diluted and evaluated using the Quant iT PicoGreen dsDNA Assay Kit.

### 2.5. Organoid Fixation, Cryosectioning and H&E (Hematoxylin and Eosin) Staining

To monitor the overall tissue morphology of the NHDF organoids, such were harvested at d1 and d14 (*n* = 3 donors) and fixed with 4% PFA/PBS and cryoprotected via sucrose gradient. Samples were embedded in Tissue-Tek (Sakura Finetek, Alphen aan den Rijn, The Netherlands), cut longitudinally using a cryotome (Leica, Nussloch, Germany) and 10 µm-thick sections were collected onto glass slides and stored at −20 °C until use. For H&E staining (chemicals from Carl Roth, Karlsruhe), sections (*n* = 3) were rehydrated in PBS for 5 min, placed in hematoxylin solution for 5 min, quickly rinsed with 0.1% HCl, washed in tap water for 5 min and counterstained in eosin for 2 min, rinsed with distilled water, and dehydrated by an ascending alcohol series. After being immersed in xylene, slides were mounted and images were taken on an upright microscope (Carl Zeiss Microscopy, Jena, Germany).

### 2.6. F-Actin Staining

To visualize the shapes and cytoskeletal organization of the cells within the organoids, F-acting staining was performed. Sections from NHDF organoids (for d1 and d14, n = 3) were permeabilized with 0.2% Triton X-100 (Sigma-Aldrich) in PBS for 10 min. Sections were then incubated with Phalloidin-iFluor 594 (Abcam, Cambridge, UK) at a 1:200 dilution in 10% goat serum (Sigma-Aldrich) for 1 h at room temperature (RT). After rinsing with PBS, slides were nuclear counterstained with 4′,6-diamidino-2-phenylindole solution (DAPI; dilution: 1:10,000; Sigma-Aldrich) for 10 min at RT. Representative fluorescence images were taken on an inverted microscope equipped with a CCD camera (Carl Zeiss).

### 2.7. Ki-67 Staining and Quantification

Ki-67 was used to analyze the abundance of proliferative cells in the organoids. Sections from NHDF organoids (for d1 and d14, *n* = 3) were permeabilized as described in Section 2.6, blocked with 10% goat serum for 1 h at RT, and incubated with a primary rabbit anti-Ki67 antibody (1:100 dilution in 10% goat serum; Abcam, Cambridge, UK) overnight at 4 °C. Slides were then washed with PBS and incubated with fluorescently-labeled goat anti-rabbit secondary antibody (1:200 dilution in 10% goat serum, Jackson ImmunoResearch, Ely, UK) for 1 h at RT. Afterwards, nuclei were stained with DAPI and slides were mounted. Representative fluorescence images were taken with the same microscope and camera as in Section 2.6. The quantitative analysis was conducted using ImageJ software according to the following algorithm: (1) 9 images per organoid were converted to grayscale; (2) thresholds were set using the automated routine “make binary” and “watershed” and applied to separate adjoining nuclei; (3) the total number of nuclei was counted automatically using the “analyze particles” tool; (4) Ki-67 positive nuclei were counted manually in the original color images; and (5) the number of Ki-67-positive nuclei were then divided by total nuclear count and the result was expressed as a percentage.

### 2.8. Terminal Deoxynucleotidyl Transferase dUTP Nick end Labeling (TUNEL) Assay

TUNEL staining was utilized to quantify the number of apoptotic cells in NHDF organoids. Sections from one representative donor (n = 1)/group/time point were subjected to an in situ Cell Death Detection Fluorescein Kit following the manufacturer’s instructions (Roche, Basel, Switzerland). In parallel, negative control sections were not exposed to the enzyme solution, whilst positive control sections were treated at the beginning with DNase I for 10 min at RT. Afterward, sections were rinsed with PBS, nuclei were counterstained with DAPI, and slides were mounted and imaged with the same microscope and camera as in Section 2.6.

### 2.9. Polymerase Chain Reaction (PCR) Analyses

Next, 2D-cultivated NHDF (n = 2) and HBTC (n = 1) as well as NHDF organoids (*n* = 3, 0 nM DEX, d14) were used for RNA isolation and follow up PCR analyses with primers for dermis- (semi-quantitative PCR) [35] and DEX-related genes (quantitative PCR), which are listed in Table 1. GAPDH (Primer ID: qHsaCEP0041396; Bio-Rad Laboratories, Hercules, CA, USA) was used as the reference gene.

Pilot quantitative PCR analyses with representative NHDF (*n* = 1 for 0 nM DEX and 10 nM DEX) and HBTC (*n* = 1) organoids were carried out with custom-designed PCR plates (Bio-Rad Laboratories) including multiple genes that are listed in Table 2. First, the RNA was extracted with the RNeasy Plus Universal Mini Kit (Qiagen, Hilden, Germany) according to the manufacturer’s instructions. For cDNA synthesis, the Transcriptor First Strand cDNA Synthesis Kit (Thermo Fischer Scientific) was used with 1 µg RNA per reaction following the manufacturer’s protocol.

PCR were performed using a Bio-Rad PCR machine with the standard program as follows: (1) 2 min at 95 °C; and (2) 40 cycles consisting of denaturation for 5 s at 95 °C, annealing/elongation steps for 30 s at 60 °C each. Semi-quantitative PCR products were analyzed on agarose gels. For quantitative PCR, the ΔΔCt method was applied to calculate relative gene expression, which was expressed as fold change. For the heat generation map, threshold levels were defined, using the Ct values of the housekeeper genes *GAPDH*, HPRT and B2M (for full name refer to Table 2) as follows: (1) high expression = Ct value lower than the one of *B2M* (Ct < 16); (2) moderate expression = Ct value in between *B2M* and *GAPDH* (16 < Ct < 24); and (3) low expression = Ct value between *GAPDH* and *HPRT1* (24 < Ct < 32); (4) not detectable/not expressed = Ct > 32.

### 2.10. Statistics

GraphPad Prism 8 software (GraphPad Software, San Diego, CA, USA) was used to express data, generate graphs, and perform statistical analyses using unpaired student’s *t*-test (each group was compared to 0 nM control). Differences were considered statistically significant when * *p* < 0.05; ** *p* < 0.01; *** *p* < 0.001 and **** *p* < 0.0001.

## 3. Results

### 3.1. NHDF Phenotypisation in 2D Culture

**Figure 1:**
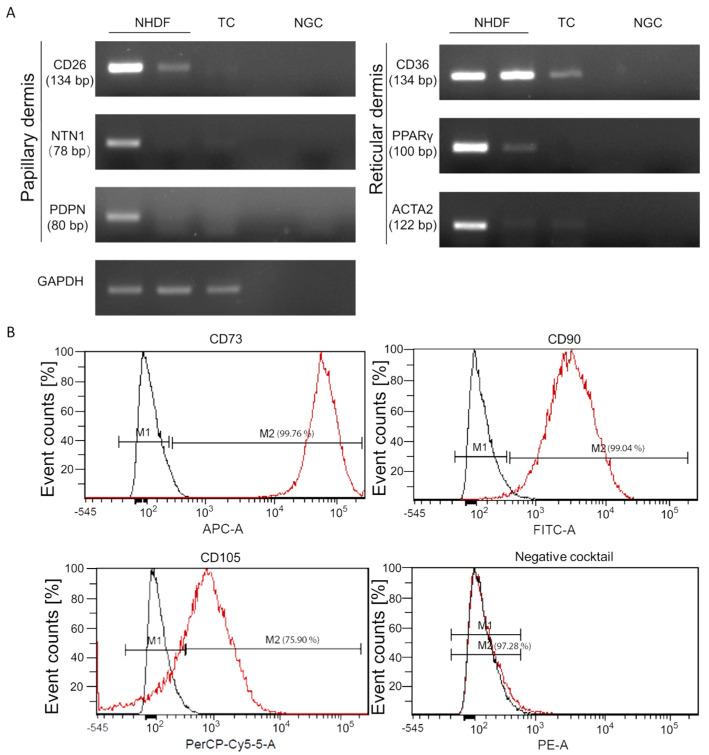
Brief characterization of commercial NHDFs. Expression analyses of (**A**) papillary and reticular dermis gene markers in normal human dermal fibroblasts (HDFs, n = 2) and tendon-derived cells (TC, n = 1) using semi-quantitative PCR analysis, and (**B**) MSCs (CD73, CD90 and CD105) and hematopoietic cell markers (negative cocktail for HLA-DR, CD11b, CD19, CD34 and CD45) in one representative NHDF donor (n = 1) by FACS.

**Figure 2:**
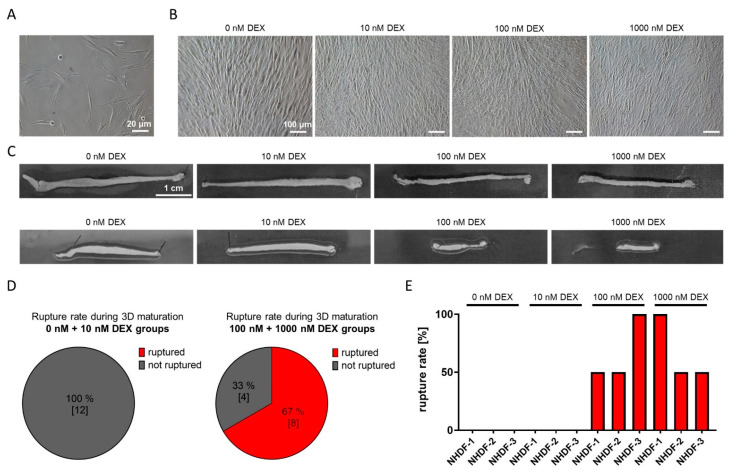
NHDF 2D expansion, 2D stimulation, 3D organoid formation and maturation. (**A**) Representative phase-contrast image of NHDFs at d1 of 2D expansion step. (**B**) Representative phase-contrast images of 0, 10, 100 and 1000 nM DEX NHDF organoids at d14 of 2D stimulation step. (**C**) Representative macroscopic images of NHDF organoids at d1 (upper panel) and d14 of 3D maturation step (lower panel). (**D**) Group-specific rupture rate over the course of 3D maturation for 0 and 10 nM DEX (to the left, two groups of data) and 100 and 1000 nM DEX organoids (to the right, two groups of data) (n = 3, 2 organoids/donor, [number] indicates total organoid number). (**E**) Donor-specific rupture rate (NHDF-1, -2, -3, indicates the individual donors).

Firstly, the commercial NHDFs were briefly validated by PCR analysis of dermis-related genes and FACS (for MSC and hematopoietic CD proteins). PCR gel electrophoresis revealed that NHDFs (*n* = 2 representative donors) expressed dermis markers, namely *CD26*, *NTN1*, *PDPN*, *CD36*, *PPARγ* and fibroblast, and myofibroblast marker *ACTA2*). As shown in Figure 1A, one of the NHDF donors showed a tendency for higher expression levels of all tested genes compared to the second donor, which could be related to the positional origin of the dermis tissue biopsy (refer to discussion) used for the isolation of the NHDFs. In contrast, TCs expressed *CD36* and *ACTA2* at low levels. FACS analysis (n = 1 representative donor) demonstrated that NHDFs were positive for CD73, CD90 and CD105, whilst negative for HLA-DR, CD11b, CD19, CD34 and CD45 (Figure 1B). In 2D expansion, NHDFs demonstrated typical fibroblast morphology of mainly bipolar or trigonal cells (Figure 2A).

### 3.2. Organoid Formation, Macroscopic Appearance and Rupture Rate of Untreated and DEX-Treated NHDF Organoids

Figure 2B shows representative phase-contrast images of confluent NHDF layers with 0, 10, 100 or 1000 nM DEX supplementation (for each n = 3) at the end of the 2D stimulation step. At this step, no obvious differences between the four study groups, as well as between individual donors, were observed. Next, cell layers were manually detached from their culture dish, rolled into a rod-like organoid (d1, Figure 2C, upper panel) and cultivated under static tension for 14 days (Figure 2C, lower panel). The formation of 3D organoids from 2D cell layers was successful in each attempt, regardless of group or donor, resulting in a success rate of 100%. At d1, 0 and 10 nM NHDF organoids of all three donors were larger and more robust than those in the 100 and 1000 nM DEX groups, which appeared thinner and more fragile. At d14 of 3D maturation, organoids displayed a glistening white appearance and were perceived as being denser than d1 organoids, indicating a contraction, as well as suggesting structural in-organoid reorganization between d1 and 14. Additionally, 100 and 1000 nM DEX-treated organoids demonstrated a strong decrease in organoid length, which was often a consequence of organoid rupture within these groups during 3D maturation. Figure 2D,E show a group- and donor-specific rupture rate during 3D maturation.

Despite the highly successful organoid formation in each study group, the frequent ruptures of the 100 and 1000 nM DEX organoids during the 3D maturation step pointed towards the inferior quality of these organoids compared to their 0 and 10 nM DEX counterparts.

### 3.3. Organoid Wet Weight and Morphological Analysis

**Figure 3:**
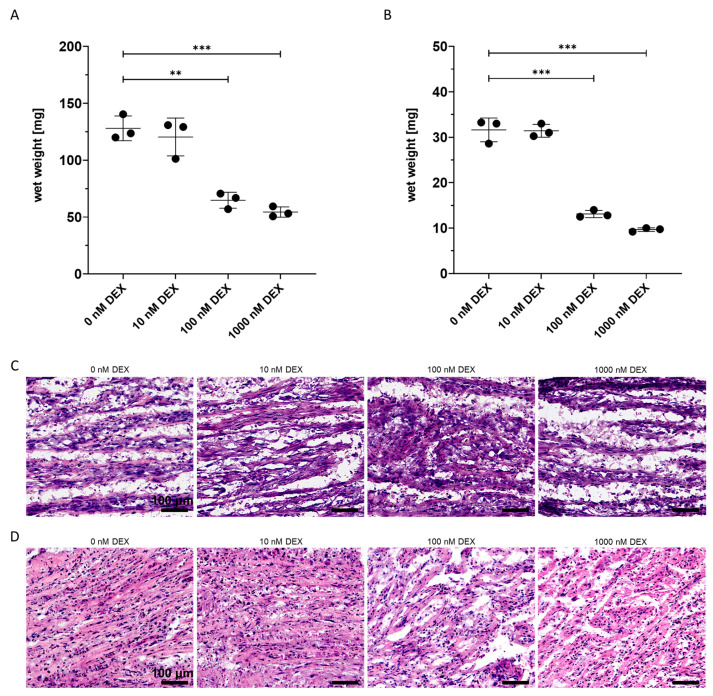
Investigation of wet weight and general morphology of NHDF organoids. (**A**) Wet weight right after organoid formation, d0. (**B**) Wet weight at the end of the 3D maturation process, d14. Lines show mean values ± SD for *n* = 3 donors and each dot indicates individual donor; ** *p* < 0.01, *** *p* < 0.001. (**C**) Representative images of H&E-stained sections of NHDF organoids at d1 of 3D maturation. (**D**) Representative images of H&E-stained sections of NHDF organoids at d14 of 3D maturation.

Organoids were weighed directly after formation (d0) and again at d14 of 3D maturation. Compared to the untreated group, 100 and 1000 nM DEX led to a significantly decreased wet weight (Figure 3A). Interestingly, this difference also remained at the end of the maturation period (Figure 3B). There was no statistical difference between the wet of untreated and 10 nM DEX-treated organoids at d0 and d14. However, a pronounced ca. 4-fold decrease in the wet weight was found between the two timepoints in all groups, suggesting marked contraction and in-organoid ECM reorganization occurring during the maturation period. To monitor the general organoid morphology, longitudinal sections of NHDF organoids were collected at d1 and d14 of the 3D maturation step and were also H&E-stained. At d1, all groups exhibited none-integral layers that were very cellular with little surrounding ECM. This finding was independent of the DEX application and dose (Figure 3C). Furthermore, within the organoid layers, cells were rarely elongated and aligned along the axis of the tensile load; most of the cell nuclei appeared round and heterogeneous in size.

**Figure 4:**
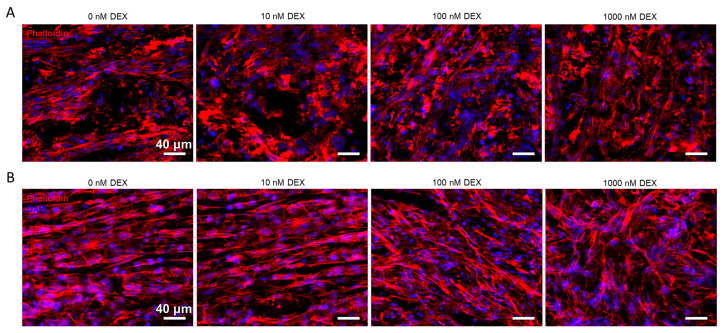
Representative fluorescent images of phalloidin- and DAPI-stained organoids at d1 (**A**) and d14 (**B**) of 3D maturation. Cell nuclei appear in blue, while F-actin in red.

In all study groups, H&E analysis of d14 organoids revealed an obvious increase in eosin-signal, suggesting ECM deposition during the 3D maturation step (Figure 3D). Clear distinctions in tissue morphology depending upon DEX concentration was observed, namely 0 and 10 nm DEX groups, which demonstrated integral layers with areas of continuous, anisotropic ECM containing aligned rows of single- or multi-strings of elongated cells. On the other hand, the groups receiving 100 and 1000 nM DEX concentrations universally exhibited a rather disorganized ECM, without cellular alignment and very variable nuclear shapes and sizes (Figure 3D). In order to closely look at how cells are organized, fluorescent imaging of organoids subjected to phalloidin-based F-actin DAPI-based nuclear staining at d1 and 14 of 3D maturation was performed (Figure 4A). Nuclei and actin filaments of one-day-old organoids of all groups were mainly in disarray, which was in line with the H&E data. However, in 14-day-old organoids from 0 and 10 nM DEX groups, cells had built well-ordered and aligned networks judged by DAPI staining. Furthermore, the cells were filled with actin filaments parallel to the organoid long axis (Figure 4B). Even though the F-actin organization of NHDFs within the 100 and 1000 nM DEX-treated organoids improved over time, it did not reach the level of alignment seen in the 0 and 10 nm DEX groups. Collectively, the above analyses revealed that the 0 and 10 nM DEX organoids were superior in tissue morphology to high DEX organoids.

### 3.4. Investigation of dsDNA Content, Cell Proliferation and Apoptosis in the Different Organoid Groups

**Figure 5:**
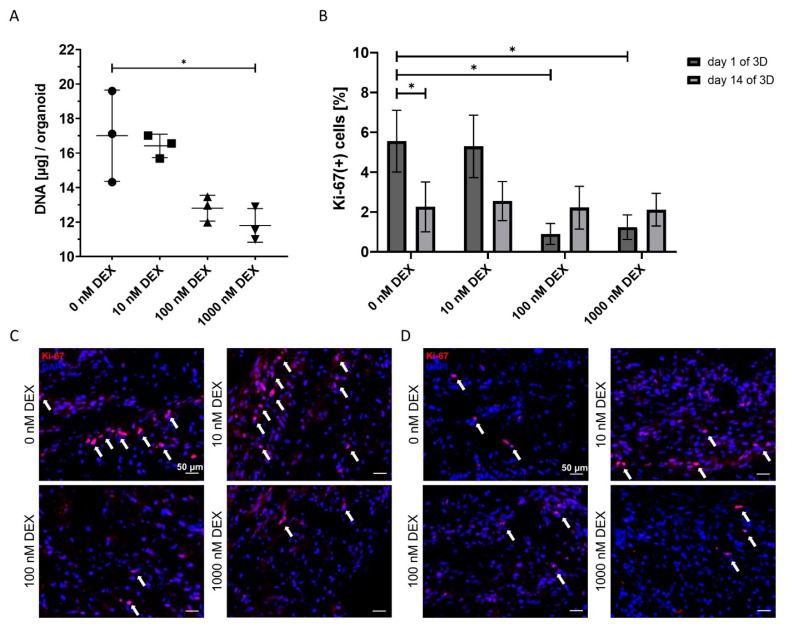
Analyses of organoid DNA content and incidence of proliferative cells. (**A**) PicoGreen-based DNA quantification directly after organoid formation (d0). Lines show mean values ± SD for *n* = 3 donors and each dot indicates individual donor; * *p* < 0.05. (**B**) Quantification at d1 and d14 of Ki-67-positive cells in percentage to DAPI-positive nuclei of all organoid groups. Bars show mean values ± SD for *n* = 3 donors; * *p* < 0.05. (**C**,**D**) Representative fluorescent images of Ki-67 staining at d1 and d14 of 3D maturation, respectively. Cell nuclei appear in blue, while Ki-67-positive cells in red. Arrows indicate single or clusters of Ki-67-positive cells.

**Figure 6:**
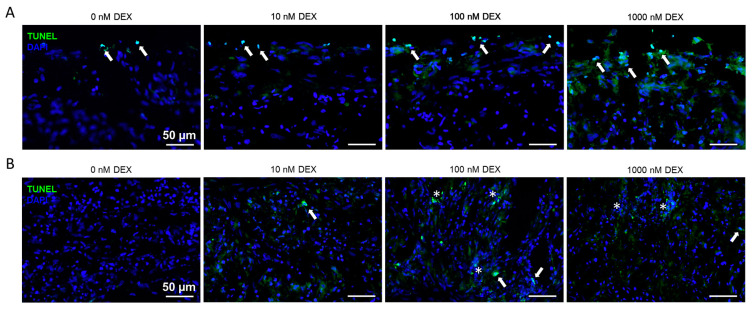
Representative images of TUNEL-stained sections of d1 (**A**) and d14 (**B**) organoids of all groups. Nuclei appear in blue, while TUNEL-positive cells in green. Arrows point still intact TUNEL-labelled nuclei, while asterisks indicate areas of more diffuse TUNEL staining related to nuclear fragmentation.

PicoGreen-based assay for dsDNA quantification was applied immediately following organoid formation (d0) to evaluate the anti-proliferative effects of DEX due to its application during the 2D stimulation step of the protocol. A significant decrease in the DNA amount was found for the 1000 nM DEX group compared to the untreated control group (Figure 5A). Such a tendency (*p*-value = 0.057) was also observed for the 100 nM DEX group. Next, cell proliferation was evaluated by quantifying Ki-67-positive cells in organoid sections of all groups at d1 and 14 of 3D maturation. At d1, the 0 and 10 nM DEX groups were comparable to one another, whereas 100 and 1000 nM DEX organoids showed a significantly lower number of proliferating cells compared to the untreated group (Figure 5B). At d14, significant differences could no longer be found between DEX-treated and untreated groups. Significant in-group change between the two different time points, was only detected for the 0 nM DEX group (Figure 5B). Figure 5C,D shows representative fluorescent images of Ki-67-stained organoids of all groups at d1 and d14, respectively. Overall, the DNA and Ki-67 results suggested that only high DEX doses lead to a significant reduction in NHDF proliferation in the organoid model. Interestingly, treatment with 100 and 1000 nM DEX correlated with fragmented DAPI-stained nuclei (Figure 5C,D and Figure 6), thereby suggesting the initiation of apoptosis. In order to elucidate on this possibility, TUNEL reaction was conducted with d1 and d14 organoid sections. The resulting data showed at both time points a tendency for a higher number of TUNEL-labelled nuclei and nuclear fragments in 100 and 1000 nM DEX organoids compared to 0 and 10 nM DEX organoids, hence validating the initiation of apoptosis due to the administration of high DEX doses.

### 3.5. Quantitative PCR Analyses of DEX- and Tendon-Related Gene Expression

**Figure 7:**
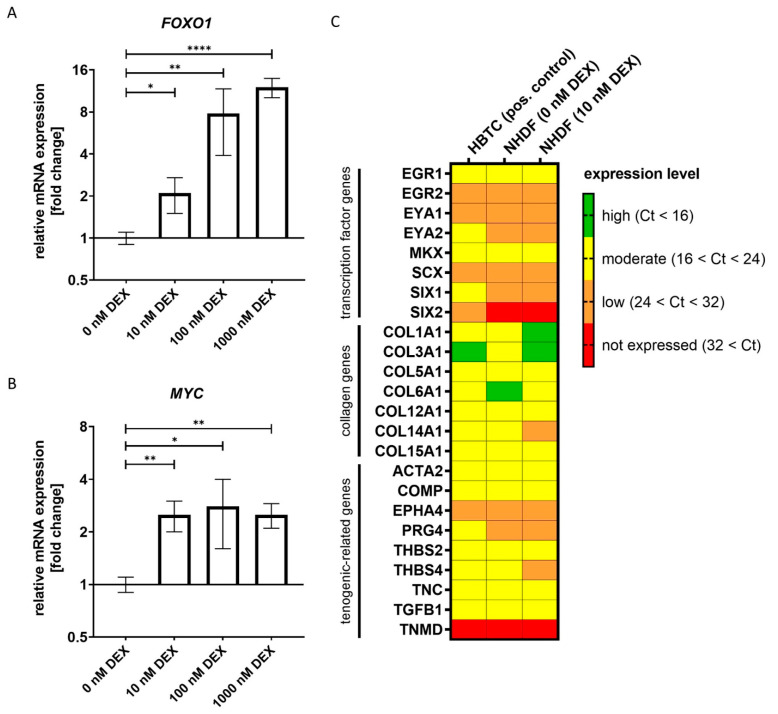
DEX- and tendon-related gene expression studies via quantitative PCR. (**A**) *FOXO1* and (**B**) *MYC* relative mRNA expression (*GAPDH* was used as reference gene) in d1 organoids of all groups. Bars show mean values ± SD for *n* = 3 donors; * *p* < 0.05, ** *p* < 0.01, **** *p* < 0.0001. (**C**) Heatmap visualizing the expression of tendon-related genes in 0 and 10 nM DEX-treated NHDF organoids in comparison to HBTC. Pilot analysis were performed at d14 of 3D maturation, *n* = 1 donor/group. Color scale indicates the expression levels based on thresholds defined by using the Ct values of the housekeeper genes *GAPDH*, *HPRT* and *B2M* as follows: (1) high expression, Ct value lower than the one of *B2M* (Ct < 16); (2) moderate expression, Ct value in between *B2M* and *GAPDH* (16 < Ct < 24); (3) low expression, Ct value between *GAPDH* and *HPRT1* (24 < Ct < 32); (4) not detectable/not expressed, Ct > 32.

Quantitative PCR was carried out using d1 NHDF organoids for two genes, whose expression is known to be affected by DEX in vitro, namely forkhead box O1 (*FOXO1*) and BHLH transcription factor, proto-oncogene *MYC* [24,36,37,38]. *FOXO1* mRNA levels were significantly upregulated in all DEX-treated groups in a dose-dependent manner. The highest fold-change of 12 was detected in the 1000 nM DEX group, followed by the 100 nM DEX group with 8-fold upregulation and, finally, the 10 nM DEX group with 2.1-fold upregulation (Figure 7A). Regarding *MYC* expression, significant upregulation was also detected in all groups compared to the untreated controls; however, no clear dose dependency was found in this case (Figure 7B). In brief, the above results demonstrated that the DEX administration during the 2D stimulation step was indeed functional, since it led to the activation of two different genes known to be downstream of DEX:

Finally, we performed a pilot screening of the expression of a number of tendon-related genes by implementing the following organoid types: (1) HBTC organoid served as the positive control; (2) 0 nM DEX-treated organoid represented the status quo for NHDFs; and (3) the 10 nM DEX organoid was chosen because such organoids exhibited the best phenotype among the DEX-treated groups. The expression analysis included 24 different genes that have been related to tendon tissue and cells (Table 1 and Figure 7C). In this pilot study, only n = 1 donor per group was evaluated due to the large number of investigated genes; the scope was to generate a guiding heatmap, which can be used as foundation for follow-up detailed analysis. Figure 7C demonstrated that apart of the transcription factor Six2 and the tendon late marker *TNMD*, the rest of genes were expressed in all three organoid types. When comparing untreated NHDF organoids to HBTC, only five genes were differentially expressed namely, *EYA2*, *SIX1*, *SIX2* and *PRG4* were downregulated, while *COL6A1* was upregulated in NHDFs. With regards to DEX application, *COL1A1* and *COL3A1* were upregulated, while *COL6A1*, *COL14A1* and *THBS4* were downregulated in 10 nM DEX organoids compared to untreated NHDF organoids. Altogether, this pilot investigation suggested a very comparable expression of multiple tendon-related genes indicative of the pro-tenogenic nature of 3D NHDFs organoids for untreated and 10 nM DEX groups.

## 4. Discussion

In this study, the dose effect of DEX on the NHDF tenogenic differentiation using self-assembly 3D organoid models was investigated with the leading hypothesis that DEX administration will regulate excessive cell proliferation and facilitate in vitro tenogenesis of this cell type. Controversial DEX effects, positive and negative, on tendon and other mesenchymal cells have been reported in the literature [24,25,26,27,28,29,30,31,32,33,39,40,41,42,43]. Despite the opposing effects described, investigating the actions of DEX is very important because it is an indispensable factor in the differentiation of mesenchyme-derived cell types. Moreover, its precise actions, dose, time and length of application on tendon cells or alternative sources for tendon differentiation, tissue engineering and therapy are still not fully understood. DEX might also be a relevant therapeutic factor, as a recent study has proposed that administering low-dose DEX might prevent secondary tendon damage during joint injury [39]. Three different doses were examined in this study, namely 10, 100 and 1000 nM DEX, and compared to untreated controls. The DEX was supplemented in the 2D stimulation step, where the cells are organized in a continuous monolayer with the purpose to subdue further cell division, as well as to moderate apical ECM deposition. To monitor the DEX effects during this step, some of the organoids were collected after rolling. The addition of DEX during the 3D maturation step was excluded for the following reasons: (1) in this step, natural cell proliferation is restricted [16,17,18], and (2) to avoid interference with TGF-β3 and suppression of ECM deposition.

Initially, a concise validation of the commercial NHDF cells was carried out by means of PCR and FACS. Then, organoids were collected at the beginning and end of the 3D maturation step and subjected to various analyses. When comparing the wet weight of NHDF organoids on d14 to the study by Yan et al. [18], where organoids formed by tendon stem/progenitor cells (TSPCs) derived from young (Y-TSPCs) and old (A-TSPCs) donors were evaluated, 0 and 10 nM DEX-treated organoids were similar to Y-TSPC organoids, while 100 and 1000 nM DEX organoids were comparable to A-TSPC organoids. In contrast to [18], where a high forming failure rate for A-TSPC organoids was reported, all NHDF groups successfully generated organoids. However, an increased fragility and an elevated rupture rate during 3D maturation was observed for the 100 and 1000 nM DEX groups. These data suggested that high DEX concentrations might be counterproductive in inducing a tendon-like phenotype in NHDFs.

General organoid morphology was assessed by H&E, F-acting and DAPI nuclear staining at two different time points. At d1 of maturation, the cell layers formed by the rolling of the organoids were not yet integral, and no differences in tissue organization were apparent between the four study groups; nonetheless, this timepoint was of interest because it could elucidate on the effect of DEX during the 2D stimulation step. In further experiments, it would be of interest to analyze the organoids between d1 and d14 in order to gain more knowledge about the structural reorganization that occurs during the maturation process. Regarding d14, a split of the study groups was noticed: 0 and 10 nM DEX-treated organoids versus 100 and 1000 nM DEX-treated organoids. Interestingly, this distinction was valid also for organoid wet weight, size and rupture rate. Cell layers remained unfused and poorly organized in 100 and 1000 nM DEX-treated organoids, as well as cell-to-ECM ratio, which appeared higher than those of the 0 and 10 nM DEX groups, which is in line with reports in the literature that DEX can negatively affect collagen production and a number of other genes [24,26,31,40,41,42,43,44,45]. Furthermore, at d14, high (100 and 1000 nM) DEX organoids exhibited isotropic ECM organization, which is typical for dermis, since this tissue type can withstand multiaxial mechanical loads [44] in contrast to tendon uniaxial stretching. Even though low (10 nM) DEX organoid morphology was superior to high DEX organoids, when compared to the morphology of Y-TSPC organoids [18], several differences were observed, namely DEX organoids still demonstrated a higher cell density and lesser cellular elongation and alignment, indicating that DEX supplementation was not sufficient to improve the overall NHDF organoid morphology.

Regarding cell quantity and proliferation within the different DEX-treated organoids, the results of two assays were in line, namely, PicoGreen-based dsDNA quantification and staining for Ki-67, a cell cycle marker [45]. The administration of DEX reduced the NHDF proliferative activity similar to other in vitro studies [23,25,26]. Specifically, the DNA amount of 0 and 10 nM DEX organoids was comparable to that of Y-TSPC organoids [18]. Significant DNA reduction was found only in 1000 nM DEX group. DEX inhibition of DNA synthesis has been correlated to a downregulation of proliferating cell nuclear antigen (PCNA) mRNA and protein levels in rat tendon fibroblasts [26]. Hence, investigating PCNA expression in DEX-treated NHDF organoids could be of interest in follow-up studies. Here, to evaluate the incidence of proliferative cells, Ki-67 staining and quantification were performed, demonstrating at d1 a higher number of Ki-67-positive cells in low versus high DEX groups, which also indicated that, as expected, DEX given during the 2D stimulation step can regulate NDHF proliferation, but only at 100 and 1000 nM. Later on, at d14, all organoid groups had a low occurrence of dividing cells and became comparable to one another. Interestingly, treatment with 100 and 1000 nM DEX resulted in fragmented DAPI-stained nuclei, thereby suggesting apoptosis. Programmed cell death via apoptosis is accompanied by nuclear shrinkage and subsequent fragmentation [46]. TUNEL staining was implemented to monitor apoptotic cells, which were more frequently detected in high DEX-treated organoids, especially at d1. In sum, our dose-dependent analyses showed that DEX can inhibit cell density and proliferation at 100 and 1000 nM, but at the same time can also lead to cell apoptosis. In sum, at d14, both groups of 0 nM DEX and 10 nM DEX organoids were qualitatively comparable, and the closeness of these groups was also visible in the quantitative DNA and Ki-67 data. Nevertheless, in follow up studies, it is of great importance to characterize in detail the microarchitecture of the organoids, their cellular organization and deposited ECM. For this, histomorphometrical analyses of the organoids should be carried out, histological and TEM data should be used, and a number of parameters (e.g., cell elongation, angular deviation, collagen fibril deposition, number, diameter, and orientation) should be evaluated.

Glucocorticoids, including DEX, regulate the expression of various genes via transrepression or transactivation and by altering mRNA stability [47,48]. Here, the effect on the expression of two downstream effectors of DEX—*FOXO1* and *MYC* was investigated by quantitative PCR, showing, especially for *FOXO1*, a very dose-dependent upregulation in DEX-treated NHDF organoids. It has also been shown in human tendon cells that 1000 nM DEX application can remarkably induce *FOXO1* expression [24]. In the same study, the authors reported that silencing *FOXO1* via siRNA mitigated the downregulation of *COL1A1* by DEX, thus suggesting that *FOXO1* plays a crucial role in the inhibition of COL1 production in DEX-treated cells. COL1 is the most dominant protein in the ECM produced by NHDFs [49]; therefore, it is likely that the less abundant ECM observed in H&E-stained sections of high DEX organoids might be due to COL1 downregulation provoked by high *FOXO1* levels in these study groups. Moreover, DEX-dependent upregulation of *FOXO1* could also provide a mechanistic explanation for the tendency for increased NHDF apoptosis observed in 100 and 1000 nM DEX-treated organoids. It has been previously reported that FOXO1 protein can act as a master switch of tumor necrosis factor alpha (TNF-α)-induced apoptosis in primary human DFs, and *FOXO1* knockdown can significantly reduce DF death in vitro [45]. Regarding the pro-proliferative MYC gene, several studies found strong transcriptional repression of *MYC* mediated by DEX, or other glucocorticoids, in various cancer cell lines as well as in vivo [36,37,50,51,52]. Thus, a decreased *MYC* expression in organoids treated with DEX was expected. Strikingly, our data showed the opposite: a significant increase in *MYC* mRNA levels in all DEX-treated groups, which was not dose-dependent. This result is in line with results from another study that suggested that the downregulation of *MYC* by DEX might not be the case in non-cancerous cells of mesenchymal origin [38]. The authors reported in adipose-derived stem cells DEX-dependent induction of *MYC*, as well as a boost in their adipogenic differentiation [38]. Hence, applying *FOXO*1 siRNA to test whether upregulation of *FOXO1* is behind the reduced ECM deposition but increased apoptosis in high DEX organoids, as well as deciphering the precise roles of MYC in our organoid model, would be highly interesting to carry out in the future.

As a pilot analysis, 0 and 10 nM DEX-treated NHDF organoids were screened for the expression of a number of tendon-related genes and compared to HBTC organoid. Then, 10 nM DEX was selected to be the only tested DEX group because the phenotype of these organoids was superior to their high DEX counterparts. The study by Chen et al. [53] has reported that DEX can inhibit the expression of SCX, a key tendon transcription factor and, thus, tendon stem cell terminal differentiation towards tenocytes. Here, the mRNA expression of seven different tendon-related transcription factors, including *SCX*, was analyzed, and, interestingly, in DF organoids, DEX application appeared not to lead to **SCX** downregulation. Articles have demonstrated that the application of DEX can also diminish the mRNA levels of *COL1A1* and *COL3A1* in 2D-cultured DFs [26,54]. On the contrary, our heatmap suggested that in the 3D organoid model, 10 nM DEX can result in *COL1A1* and *COL3A1* upregulation. However, this finding should also be validated at the protein level in a follow up study. Moreover, the differences in DEX effects might be related to cell type, DEX dose, time and length of application, differentiation status of the treated cells, and in vitro culture model (2D, 3D). *TNMD*, a well-accepted tendon marker gene, was not detected in either of the groups which is comparable to other studies reporting a rapid loss of *TNMD* mRNA in vitro [18,55,56]. Nevertheless, it is important to note that the 3D culture itself was not sufficient to reconstitute *TNMD* expression proposing that further optimization of the organoid model is required and possible ways ahead are discussed below. Interestingly, the heatmap suggested that the majority of other investigated genes were comparably expressed in HBTC and NHDF organoids, which is a very encouraging finding on the pro- tenogenic potential of DFs. Using the heatmap prediction, follow up studies showed focus on specific gene targets and investigate donor variability and significant differences.

The present findings suggest that the administration of DEX alone, in any of the tested concentrations, is not sufficient to facilitate the tenogenic differentiation of DFs in the 3D organoid model. The DEX doses of 10, 100 and 1000 nM were chosen because they had been shown to influence cell proliferation without exhibiting cytotoxicity [24,31,32,33]. In differentiation protocols, DEX is mostly applied at a concertation of 100 nM or higher; therefore, in this study, 10 nM was selected as the lowest dose and the dose range was designed in consecutive 10-fold increases. However, lower concentrations such as 1 or 5 nM might exert an effect, which has to be taken into account in follow up studies. In addition, it has been reported that DEX action can be disturbed by homologous downregulation of the glucocorticoid receptor due to prolonged administration of DEX [57]; hence, in the future, it will be important to also monitor the receptor levels in the course of the differentiation protocol.

The results of this study suggest that similar to chondrogenic differentiation, tenogenic differentiation requires more complex stimulation. Therefore, a multifactorial strategy to further optimize the protocol should be pursued in subsequent studies. Over the years, it has become better understood that DFs are a highly heterogeneous population with functional variabilities. For example, it has been reported that significant phenotypical and functional differences exist between DFs derived from different anatomic sites as well as dermis sub-layers [58,59,60]. Sacco et al. [22] reported that human DFs originating from skin of the abdomen, arm, neck, breast and thigh can differ in the expression of mesenchymal markers, proliferation index, swiftness to differentiate into adipocytes, chondrocytes and osteoblasts, as well as in secretion of various growth factors. Due to this functional DF heterogeneity, it can be speculated that the site of DF origin might be critical for tendon tissue engineering when utilizing this cell type; hence, it is of utmost importance to identify and characterize the most suitable subset of DFs to be used for further exploration. Next, the supplementation of DEX at the 2D stimulation stage and TGF-β3 at the 3D maturation stage of our protocol was not sufficient enough to enhance the performance of DFs; therefore, in follow up studies, in addition to examining further DEX doses and monitoring the receptor levels, the effect of low serum combined with ITS (insulin-transferrin-selen), as well as additional stimuli, e.g., GDF-5, -6, and FGF-2, should be tested. These growth factors have been shown to boost tenogenic differentiation and to upregulate key tenogenic genes such as SCX, MKX and TNMD in BMSCs and TSPCs [61,62,63,64,65,66,67]; hence, they could emerge as promising candidates for guiding DFs into building tendon-like tissue. Finally, examining different approaches for applying mechanical strain (e.g., static vs. dynamic) could also lead to an upgrade of the present protocol. For instance, dynamic mechanical stimulation has been shown to be more beneficial compared to static strain, presumably because it more closely resembles the conditions of in vivo tenogenesis [21]. Taken together, the above multifactorial strategy could lead to the development of a sophisticated protocol for DF-based generation of tendon-like tissues, which could be then thoroughly examined in preclinical settings to assess novel and effective treatment options for tendon injuries.

## 5. Conclusions

In this study, the effect of the dose-dependent application of DEX on the tenogenic differentiation of DFs using a previously established 3D organoid model was evaluated. The novel results demonstrated that DEX, in all tested concentrations, was not sufficient to notably induce the tenogenic differentiation of human DFs. DEX-treated organoids did not exhibit clear advantages over untreated, control organoids. Moreover, high concentrations of DEX exerted a negative impact on the organoid phenotype. Interestingly, the pilot screening on the expression of tendon-related genes in untreated and 10 nM DEX-treated DF organoids showed a largely comparable profile to organoids formed by tendon-derived cells, which is encouraging for further investigations on the suitability of DF for tendon tissue engineering and therapy. In the future, a multifactorial strategy for protocol optimization: evaluating more DEX concentrations, monitoring glucocorticoid receptor levels over time, and combining low serum with ITS, applying additional growth factors and dynamic mechanical stimulation, should be pursued.

## Figures and Tables

**Table 1:** PCR primers and annealing temperature for dexamethasone- and dermis-related genes.

Target Gene	Abbreviation	Primers	Annealing Temperature
Dipeptidyl peptidase 4	*CD26*	F 5′-TACAAAAGTGACATGCCTCAGTT-3′ R 5′-TGTGTAGAGTATAGAGGGGCAGA-3′	60 °C
Netrin 1	*NTN1*	F 5′-ACAACCCGCACAACCTGAC-3′ R 5′-GGGACAGTGTGAGCGTGAC-3′	60 °C
Podoplanin	*PDPN*	F 5′-GTGTAACAGGCATTCGCATCG-3′ R 5′-TGTGGCGCTTGGACTTTGT-3′	60 °C
CD36	*CD36*	F 5′-CTTTGGCTTAATGAGACTGGGAC-3′ R 5′-GCAACAAACATCACCACACCA-3′	60 °C
Actin alpha 2	*ACTA2*	F 5′-CTATGAGGGCTATGCCTTGCC-3′ R 5′-GCTCAGCAGTAGTAACGAAGGA-3′	60 °C
Peroxisome proliferator activated receptor gamma	*PPARγ*	F 5′-ACCAAAGTGCAATCAAAGTGGA-3′ R 5′-ATGAGGGAGTTGGAAGGCTCT-3′	60 °C
Forkhead box O1	*FOXO1*	F 5′-GGATGTGCATTCTATGGTGTACC-3′ R 5′-TTTCGGGATTGCTTATCTCAGAC-3′	60 °C
MYC proto-oncogene, BHLH transcription factor	*MYC*	F 5′-TCCCTCCACTCGGAAGGAC-3′ R 5′-CTGGTGCATTTTCGGTTGTTG-3′	60 °C

**Table 2:** Gene list in custom-designed real-time PCR plates.

Target Gene	Abbreviation	Category
Early growth response 1	*EGR1*	Tendon transcription factor gene
Early growth response 2	*EGR2*	Tendon transcription factor gene
Eyes absent homolog 1	*EYA1*	Tendon transcription factor gene
Eyes absent homolog 2	*EYA2*	Tendon transcription factor gene
Mohawk homeobox	*MKX*	Tendon transcription factor gene
Scleraxis	*SCX*	Tendon transcription factor gene
SIX homeobox 1	*SIX1*	Tendon transcription factor gene
SIX homeobox 2	*SIX2*	Tendon transcription factor gene
Collagen type I, alpha 1	*COL1A1*	Collagen gene
Collagen type III, alpha 1	*COL3A1*	Collagen gene
Collagen type V, alpha 1	*COL5A1*	Collagen gene
Collagen type VI, alpha 1	*COL6A1*	Collagen gene
Collagen type XII, alpha 1	*COL12A1*	Collagen gene
Collagen type XIV, alpha 1	*COL14A1*	Collagen gene
Collagen type XV, alpha 1	*COL15A1*	Collagen gene
Actin alpha 2 (α-smooth muscle actin)	*ACTA2*	Fibroblast-, myofibroblast-related gene
Cartilage oligomeric matrix protein	*COMP*	Tendon-related gene
Ephrin type A receptor 4	*EPHA4*	Tendon-related gene
Proteoglycan 4	*PRG4*	Tendon-related gene
Thrombospondin 2	*THBS2*	Tendon-related gene
Thrombospondin 4	*THBS4*	Tendon-related gene
Tenascin C	*TNC*	Tendon-related gene
Transforming growth factor beta 1	*TGFB1*	Tendon-related gene
Tenomodulin	*TNMD*	Tendon-related gene
Glyceraldehyde 3-phosphate dehydrogenase	*GAPDH*	Housekeeper gene
Hypoxanthine-guanine phosphoribosyltransferase 1	*HPRT1*	Housekeeper gene
Beta-2 microglobulin	*B2M*	Housekeeper gene

## Data Availability

The datasets generated for this study are available on request to the first author (niklas.kroner-weigl@stud.uni-regensburg.de).
